# Comparison of immediate vs. delayed guided tissue regeneration in Infrabony defect of second molars after adjacent third molar extraction: a retrospective study

**DOI:** 10.1186/s12903-024-04591-1

**Published:** 2024-07-23

**Authors:** Si-Min Tang, Di-Xin Liu, Zi-Yun Xiong, Yi-Qian Shao, Jing Jiang, Li Chen, Qin Xiong, Shuo-Yan Wu, Dong-Ying Xuan

**Affiliations:** 1 College of Dentistry, Zhejiang Chinese Medical University, Hangzhou, China; 2 Department of Periodontology, Hangzhou Stomatology Hospital, 1 Pinghai Road, Hangzhou, Zhejiang Province China

**Keywords:** Bone regeneration, Guided tissue regeneration, Molar, second, Molar, third

## Abstract

**Background:**

The distal aspect of the second molar (d-M2) often exhibits infrabony defects due to the adjacent third molar. Although the defects can be treated by guided tissue regeneration (GTR) after removing the third molar, the optimal timing remains uncertain following third molar removal in clinical decision-making. This study aimed to compare delayed and immediate GTR treatments to assist in clinical decision-making.

**Methods:**

D-M2 infrabony defects with a minimum 1-year follow-up were collected and divided into three groups: Immediate GTR group, which underwent third molar extraction and received GTR simultaneously; Delayed GTR group, which underwent delayed GTR at least 3 months after third molar extraction; and Control group, which underwent only scaling and root planing during third molar extraction. The clinical and radiographic parameters related to the infrabony defect before GTR and post-surgery were evaluated using the Kruskal-Wallis test or one-way ANOVA, followed by post-hoc Dunn’s test or the Bonferroni test for pairwise comparisons.

**Results:**

A total of 109 d-M2 infrabony defects were assessed. No significant differences were found between the two GTR groups, although both of them showed significant reductions in infrabony defect depth: the immediate GTR group (2.77 ± 1.97 mm vs. 0.68 ± 1.03 mm, *p* < 0.001) and the delayed GTR group (2.98 ± 1.08 mm vs. 0.68 ± 1.03 mm, *p* < 0.001) compared to the control group.

**Conclusion:**

GTR can effectively improve d-M2 infrabony defects when the third molar is removed, whether simultaneously or delayed. Patients may experience less discomfort with immediate GTR treatment as it requires only one surgery.

## Introduction

The presence of a distal aspect of the second molar (d-M2) infrabony defect is often the result of the adjacent third molar’s influence [1]. Research indicates that individuals with third molars are twice as likely to develop such defects at d-M2 compared to those without third molars [2]. It is important to note that a d-M2 infrabony defect does not fully heal spontaneously after third molar (M3) extraction. A 2-year retrospective study reported that 43.3% of sites where M3 was removed had pocket depths exceeding 7 mm, while 32.1% exhibited an infrabony defect depth (IBD) exceeding 4 mm [3]. Considering the second molar’s critical role in the dental arch, mastication, and occlusal stability, its significance is profound. The presence of an infrabony defect affecting the second molar’s structural integrity can significantly impact oral health and the patient’s quality of life [4].

Several approaches have been proposed to address this concern, including scaling and root planing (SRP), the distal wedge procedure, and guided tissue regeneration (GTR) [5–8]. Extensive evidence supports the effectiveness of GTR in managing d-M2 infrabony defects, with significant improvements in clinical attachment level, reduced pocket depth (PD), and bone regeneration [9–14].

When considering GTR treatment for d-M2 infrabony defects, the procedure can be performed either immediately alongside M3 extraction or delayed. Immediate GTR treatment involves a single surgical procedure, reducing the need for secondary surgery. Existing studies have focused on the effect of immediate GTR treatment, selectively choosing M3s that are entirely covered by soft tissue [5, 15–17]. It is noteworthy, however, that almost two-thirds of M3s were erupted, and among the residual impacted M3s, 81% were partially covered by soft tissue [18, 19]. Performing immediate GTR in these cases carries a high risk of membrane exposure, which influences the effectiveness of GTR treatment [20].

In contrast to immediate GTR treatment, delayed GTR treatment allows for sufficient soft tissue formation, reducing the risk of membrane exposure, and enhances GTR’s overall effectiveness [21]. To date, clinical evidence regarding delayed GTR treatment in d-M2 has been scarce, and there are no studies comparing the effectiveness of delayed and immediate GTR treatments.

Moreover, many factors affect the effectiveness of GTR. Patient-related factors, defect morphology, and surgical techniques have all been reported to significantly impact the overall predictability of the GTR procedure [22]. A recent study reported that d-M2 infrabony defects occur in both the mandible and maxilla, with maxillary d-M2s more likely to have deeper PD than mandibular d-M2s [23]. Due to differences in bone density, anatomical structure, and blood supply, the location of the M2 may affect the effectiveness of GTR [24–26]. However, recent studies have focused only on the effects of different materials on GTR outcomes in d-M2, with no studies exploring the prognostic factors affecting these outcomes.

Therefore, the aim of the present study was to compare the effectiveness of delayed GTR treatment and immediate GTR treatment in managing d-M2 infrabony defects, and to assess potential prognostic factors that can affect the clinical and radiographic outcomes.

## Materials and methods

### Study design

This retrospective study included patients who received periodontal therapy at the Periodontology Department of Hangzhou Dental Hospital from January 2015 to September 2022. All paper files and digital charts of patients with infrabony defects on the d-M2 were meticulously scanned and analyzed by two independent and pre-calibrated investigators (S.-M.T., Y.-Q.S.). At each stage, upon examining the gathered data, in case of disagreement, discussions were held by the two reviewers. If resolution was not possible, a third investigator (D.-Y.X.) was consulted to reach a consensus. Approval was obtained from the institutional review board of Hangzhou Dental Hospital in Hangzhou, Zhejiang, China (approval no. 2023LL06). No significant changes were made to the trial design after the study commenced.

### Inclusion and exclusion criteria

Patients who met the following inclusion criteria were included in this study:

1. The patient’s age was > 18 years.
2. The preoperative criteria includes a PD of ≥ 6 mm on the d-M2 or a PD of ≥ 4 mm with bleeding on probing (BOP) present. Additionally, the IBD on the d-M2 was > 3 mm [27].
3. The patient’s records encompassed a 1-year follow-up after the operation.

Meanwhile, the following criteria were used to exclude patients from the study:

1. The second molar (M2) had a large amalgam restoration or a distal tooth defect below the cemento-enamel junction.
2. GTR treatment was performed for another reason, not to address the infrabony defect at d-M2.
3. The M2 had a hopeless prognosis [28].
4. The patient has not received professional oral hygiene instructions in the last 6 months, or the patient’s FMPS > 25% before the surgery [29].
5. The patient is medically compromised, including those on medication affecting healing or patients with uncontrolled diabetes, blood dyscrasias, substance abuse, heavy smoking, or acute infections.

### Data collection and classification

The following data were collected from all eligible participants:

1. Patient-related factors: age and sex.
2. Medical history: documented smoking, diabetes, and other systemic or local diseases.
3. Location of the treated defect: mandible or maxilla.
4. Relevant clinical parameters: specifically PD.
5. Patient’s cone beam computed tomography (CBCT) records.
6. Infrabony defect morphology was assessed using the Goldman et al. classification, with defects defined as having one osseous wall, two osseous walls, three osseous walls, or a combination of defects [30], based on the latest CBCT data before GTR treatment.

### Group classifications

Patients were classified into one of three groups for the present study. Immediate GTR group included patients presenting with infrabony defects in the d-M2 region, who underwent immediate GTR treatment during extraction of M3. Delayed GTR group included patients presenting with infrabony defects in the d-M2 region, who underwent delayed GTR treatment ≥ 3 months after extraction of the adjacent M3. Finally, the control group included patients presenting with infrabony defects in the d-M2 region, who only underwent SRP, without any GTR intervention.

### Surgical procedure

After administering local anesthesia, a full-thickness mucoperiosteal flap was raised in the immediate GTR group. Flap elevation occurred in the delayed GTR and control groups as required. Gentle extraction of M3 was performed, along with osteotomy and odontotomy based on the impaction status. Following extraction, the residual bone cavity underwent curettage. Subsequently, manual instruments were used to perform SRP on the adjacent M2’s distal surface.

In the immediate GTR group, the d-M2 infrabony defect was filled with deproteinized bovine bone mineral (Bio-Oss^®^; Geistlich Pharma AG, Wolhusen, Switzerland) under gentle pressure. A collagen membrane (Bio-Gide^®^; Geistlich Pharma AG) was placed over the bone substitute, trimmed to cover the defect 4 mm beyond the buccal and lingual defects, and firmly adapted to prevent mobility. Mattress and interrupted sutures (Ethilon^®^ 4 − 0; J&J MedTech, New Brunswick, NJ, USA) (Prolene^®^ 4 − 0; J&J MedTech, New Brunswick, NJ, USA) were then applied to stabilize the material and immobilize the gingival tissue. The suture material type was randomized and chosen by the surgeon.

The patients were given amoxicillin plus clavulanic acid, 1 g, every 12 h for 5 days, and ibuprofen was prescribed as an analgesic (600 mg twice a day) for 4 days beginning on the day of surgery.

**Fig. 1:**
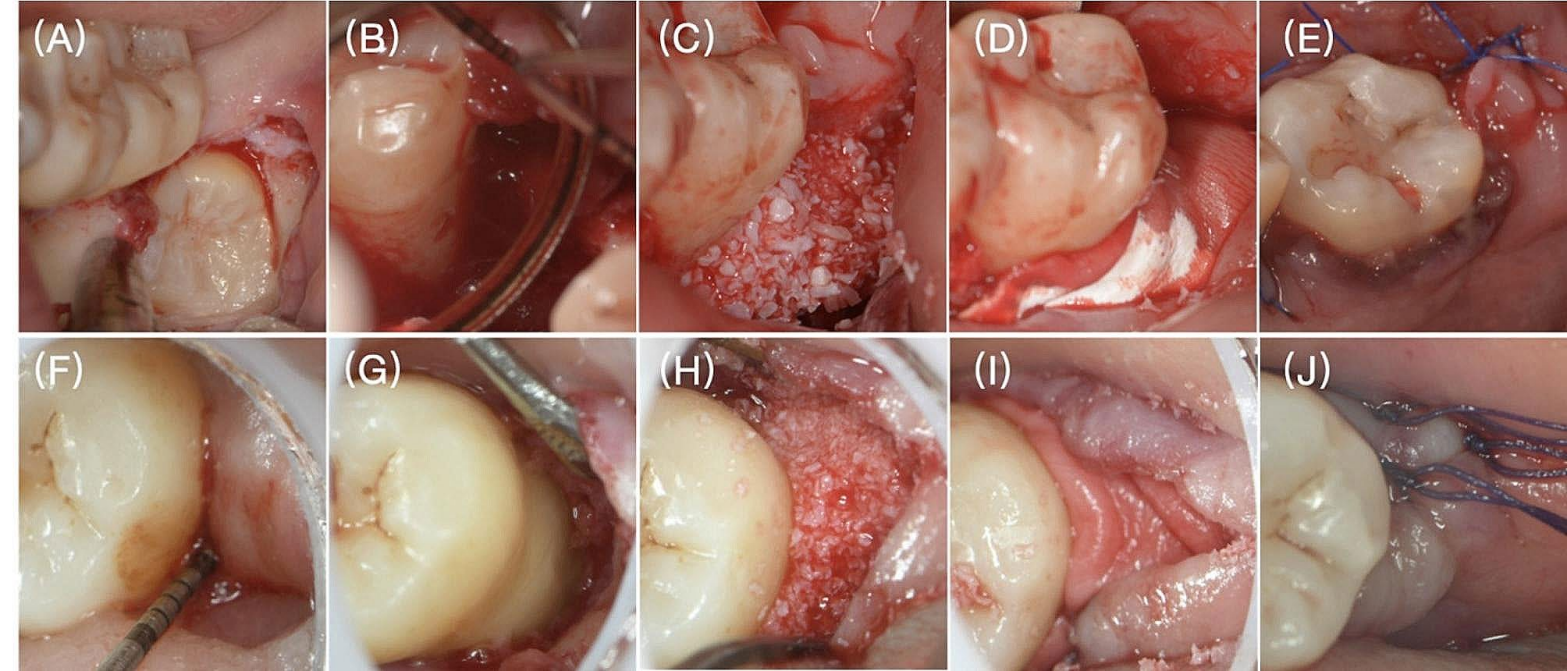
Surgical procedure: (**A**) Third molar extraction in Immediate GTR group; (**B**) Infrabony defect on the distal aspect of the second molar (d-M2) in Immediate GTR group; (**C**) Deproteinized bovine bone material (DBBM) grafted to the d-M2 infrabony defect in Immediate GTR group; (**D**) Collagen membrane (CM) placed over the bone substitute in Immediate GTR group 1; (**E**) Suture; (**F**) Pre-operative probing depth of 9 mm in Delayed GTR group; (**G**) d-M2 infrabony defect in Delayed GTR group; (**H**) DBBM grafted to the d-M2 infrabony defect in Delayed GTR group; (**I**) CM placed over the bone substitute in Delayed GTR group; (**J**) Suture.

Patients in the delayed GTR group underwent GTR ≥ 3 months after M3 extraction. Following the application of local anesthesia, a full-thickness mucoperiosteal flap was raised to expose the d-M2 infrabony defect. The residual GTR procedure and graft material were the same as used in the immediate GTR group. Surgical procedures for the GTR groups are depicted in Fig. 1.

In the control group, only SRP was performed after M3 extraction .

In all cases, sutures were removed after 14 days. Postoperatively, each patient received a 3-day antibiotic course and was instructed to use 0.20% chlorhexidine gluconate mouthwash twice daily for 2 weeks.

### Study outcomes

#### Primary outcomes

**Fig. 2:**
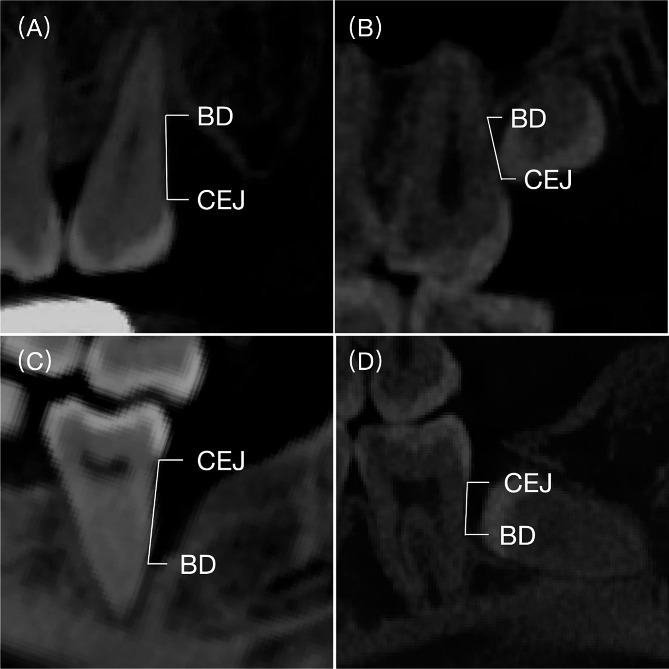
Infrabony defect depth was measured from the cemento-enamel junction (CEJ) to the base of the defect (BD) on the distal aspect of the second molar. (**A**) Maxillary second molar (M2) without adjacent third molar (a-M3); (**B**) Maxillary M2 with a-M3; (**C**) Mandibular M2 without a-M3; (**D**) Mandibular M2 with a-M3.

**Fig. 3:**
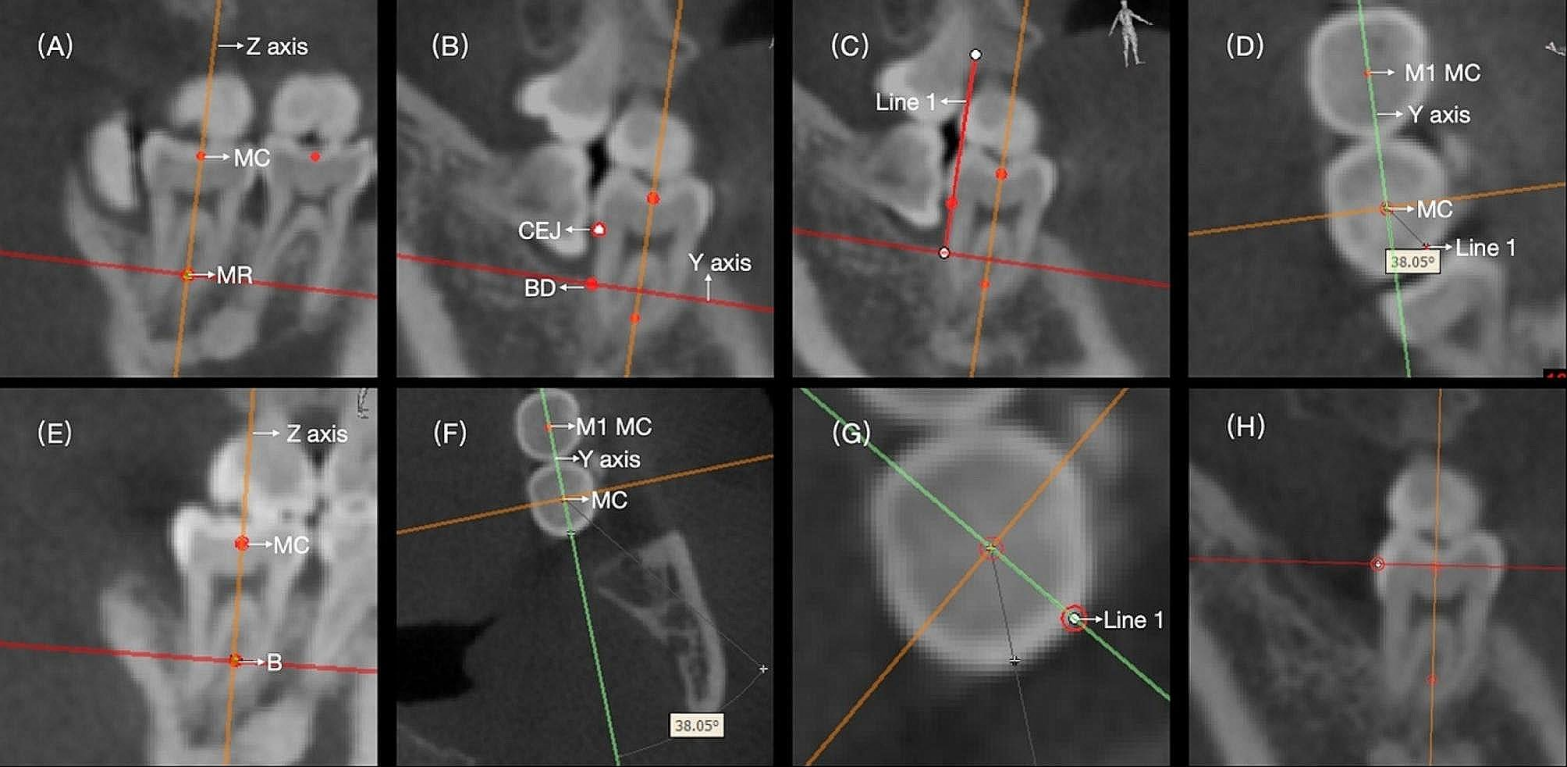
To establish a consistent measurement plane: (**A**) Verify that the Z-axis intersects the central axis of M2 on the CBCT before baseline. Define the central axis of M2 by a line passing through the midpoint (MC) of the mesiodistal crown width and a point one-third of the distance from the apex of the mesial and distal roots. (**B**) Locate the base of the defect (BD) and place it on the plane intersecting BD and the central axis (Z-axis). This plane is the measurement plane. (**C**) In the measurement plane, create a line (Line 1). (**D**) Return to the transverse plane at point MC. Line 1 should correspond to a point within this plane. Rotate the Y-axis across the midpoint of the mesiodistal crown width of M1 and measure the angle formed by Line 1, point MC, and the Y-axis. (**E**) Locate the Z-axis on the central axis at the 1-year post-operative CBCT. (**F**) Create an angle identical to the one formed by Line 1, point MC, and the Y-axis. (**G**) This final step determines the measurement plane on the post-operative CBCT

IBD changes were assessed from baseline to the 12-month after GTR surgery, and in the control group, the IBD changes were assessed from baseline to the 12-month after M3 extraction. The examination was conducted using CBCT measurements. IBD was determined by measuring the distance between the cemento-enamel junction and the base of the defect on the d-M2 (Fig. 2). This measurement was conducted based on the three-dimensional reconstruction obtained with Mimics Software (version 19.0; Materialise Mimics Medical, Belgium) [31, 32]. To ensure consistent measurements within the same scan, we first reset the Multi-Planar Reconstruction (MPR) plane and verify that the Z-axis intersects with the central axis of M2. The central axis of M2 is established by passing through the midpoint (MC) of the mesiodistal crown width and the midpoint located one-third of the way from the apex of the mesial and distal roots [33]. Additionally, we identify the intersection point of the X-axis and Y-axis along the central axis of M2. Subsequently, we locate the base of the defect (BD) and mark it accordingly, rotate the Y-axis, and generate a new plane intersecting the point BD and the central axis of M2. After identifying the Cemento-Enamel Junction (CEJ) point on this plane, we draw Line 1 connecting BD and CEJ. Upon returning to the transverse plane at point MC, Line 1 corresponds to a point within this plane. Further, we rotate the Y-axis across the midpoint of the mesiodistal crown width of M1 and measure the angle formed by Line 1, point MC, and the Y-axis. This angle helps establish the IBD measurement plane at different time points (Fig. 3). The remeasurement was performed after 2 weeks for intra-reliability estimation.

#### Secondary outcomes

Treatment outcomes were assessed by comparing changes in probing depth (PD) between baseline and after GTR surgery. In the control group, the IBD changes were assessed from baseline to 12 months after M3 extraction. Measurements were taken at two specific sites on the second molar, located at the disto-buccal and disto-lingual positions, using a PCP-UNC-15 probe and recorded to the nearest 1 mm [34].

### Statistical analysis

The acquired data were input into predefined spreadsheets by the same investigator, S.-M.T. Data processing and analysis were completed using R version 4.3.0 (2023-04-21; R Foundation for Statistical Computing, Vienna, Austria). Descriptive statistics were employed to present the baseline data, and we report mean ± standard deviation values as well as ranges. The treated defects served as the unit of analysis. The changes in clinical and radiographic parameters from baseline to the 1-year outcome were assessed with dependent t-tests. Statistically significant differences among the three groups were assessed using the Kruskal-Wallis test or one-way ANOVA, followed by post-hoc Dunn’s test or the Bonferroni test for pairwise comparisons. The chi-square test was conducted to identify the correlation between defect morphology and occlusion.

Mixed-effects uni- and multi-level regression analyses were conducted to identify prognostic factors for bone fill. A significance threshold of *p* < 0.05 was adopted for statistical assessment.

## Results

### Study population

A total of 273 patients with 364 sites had their charts retrieved and screened subsequent to the initial search. A total of 255 sites were subsequently excluded for different reasons, including the insufficiency of required chart information, clinical data, or CBCT data (*n* = 116); loss during the follow-up period (*n* = 36); a follow-up duration of < 1 year (*n* = 35); retention of adjacent M3s (*n* = 29); an infrabony defect depth of < 3 mm (*n* = 18); regeneration attempts in furcation defects (*n* = 13); open flap surgery (*n* = 5); and the presence of systemic disease (*n* = 3).

Consequently, a total of 83 patients (45 women and 38 men) with 109 sites remained for the final analysis. The mean age among the study participants was 40.00 ± 8.26 years. The Immediate GTR group included 21 patients (12 women and 9 men) with a mean age of 39.05 ± 5.38 years and 23 GTR-treated d-M2 infrabony defects. The Delayed GTR group included 25 patients (13 women and 12 men) with a mean age of 38.56 ± 8.30 years and 34 GTR-treated d-M2 infrabony defects. Finally, the Control group included 37 patients (20 women and 17 men) with a mean age of 41.51 ± 9.44 years and 52 SRP-treated d-M2 infrabony defects. Detailed characteristics of the allocated patients are presented in Table 1.

**Table 1:** Baseline characteristics of the included patients/defects

Variable/Group	Level	Overall	Control	Immediate GTR Group	Delayed GTR Group	*p*
*n*		109	52	23	34	
Age		40.00 ± 8.26	41.51 ± 9.44	39.05 ± 5.38	38.56 ± 8.30	NS (0.323)
Initial infrabony defect depth [IBD (mm)]		6.93 ± 1.97	6.66 ± 1.90	7.09 ± 1.86	7.22 ± 2.14	NS (0.389)
Initial pocket depth [PD (mm)]		7.54 ± 1.61	7.33 ± 1.65	7.74 ± 1.60	7.74 ± 1.56	NS (0.419)
Occlusion (*n*, %)	Maxilla	35 (32.1)	14 (26.9)	7 (30.4)	14 (41.2)	NS (0.377)
	Mandible	74 (67.9)	38 (73.1)	16 (69.6)	20 (58.8)	
Sex (*n*, %)	Male	38 (45.8)	17 (45.9)	9 (42.9)	12 (48.0)	NS (0.941)
	Female	45 (54.2)	20 (54.1)	12 (57.1)	13 (52.0)	
Defect morphology(*n*, %)	Three osseous walls	36 (33.03)	17 (32.69)	8 (34.78)	11 (32.35)	NS(0.771)
	Combination	33 (30.28)	13 (25.00)	8 (34.78)	12 (35.29)	
	Two osseous walls	40 (36.70)	22 (42.31)	7 (30.43)	11 (32.35)	

Data are expressed as mean ± standard deviation values or numbers with percentages

### CBCT analysis

**Fig. 4:**
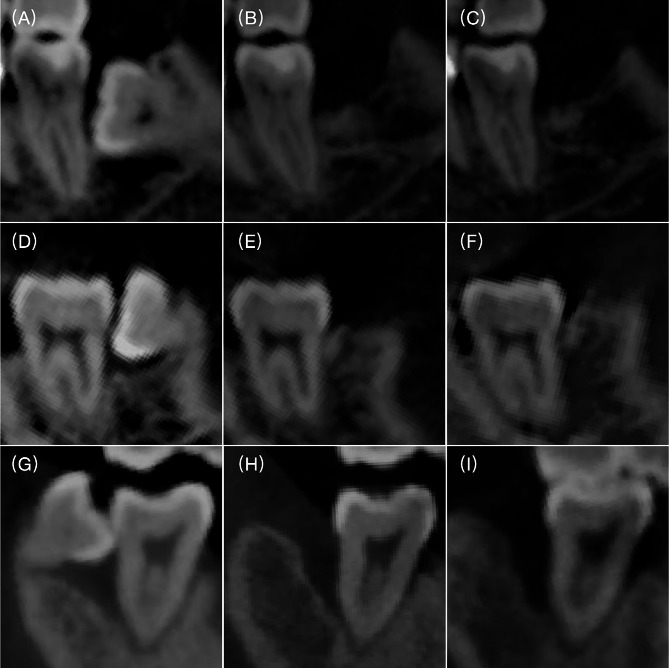
CBCT reconstruction images of the distal-buccal aspect of the second molar. (**A**) Pre-operation in Immediate GTR group; (**B**) Postoperative 6-month image in Immediate GTR group; (**C**) Postoperative 12-month image in Immediate GTR group. (**D**) Pre-operation in Delayed GTR group; (**E**) Postoperative 6-month image in Delayed GTR group; (**F**) Postoperative 12-month image in Delayed GTR group. (**G**) Pre-operation in the control group; (**H**) Postoperative 6-month image in the control group; (**I**) Postoperative 12-month image in the control group

No hard tissue abnormalities were found during the radiographic assessment. Both groups exhibited statistically significant bone regeneration after a 1-year follow-up (Table 2). In the Immediate GTR group, bone regeneration measured 2.77 ± 1.97 mm during the 1-year post-operation recall. Meanwhile, the Delayed GTR group had a measurement of 2.98 ± 1.08 mm. These values showed no statistically significant difference in reduction in IBD between the two groups (*p* = 0.376). Notably, however, the control group exhibited significantly less bone regeneration (0.68 ± 1.03 mm) compared to the GTR groups. Representative radiographic images for both groups over time were presented in Fig. 4.

**Table 2:** IBD variables of the control and GTR groups at 1 year after surgery

Parameter	Immediate GTR Group *n* = 23	Delayed GTR Group *n* = 34	Control Group *n* = 52	*p*
IBD (mm)				
Baseline	7.09 ± 1.86	7.22 ± 2.14	6.66 ± 1.90	0.389
12 months	4.32 ± 1.39	4.25 ± 1.30	5.97 ± 1.86	**< 0.001**
Reduction in IBD (mm)	2.77 ± 1.97	2.98 ± 1.98	0.68 ± 1.03	**< 0.001**
*p*-values for intergroup difference (reduction in IBD)	0.376 vs. delayed GTR **0.001 vs. control**	0.376 vs. delayed GTR **< 0.001 vs. control**	**< 0.001 vs. immediate GTR** **< 0.001 vs. delayed GTR**	

Bold signifies statistical significance. n = sites; IBD: infrabony defect depth

### Clinical analysis

Significant clinical improvements were observed in all groups, as indicated by reductions in PD from baseline. There was no significant difference in PD reduction between the Immediate GTR group (4.13 ± 1.66 mm) and the Delayed GTR group (3.47 ± 1.73 mm) (*p* = 0.223). However, the control group exhibited significantly less PD reduction (2.21 ± 2.27 mm) compared to either GTR group (Table 3).

**Table 3:** PD variables of the control and GTR groups at 1 year after surgery

Parameter	Immediate GTR Group *n* = 23	Delayed GTR Group *n* = 34	Control Group *n* = 52	*p*
PD (mm)				
Baseline	7.74 ± 1.60	7.74 ± 1.56	7.27 ± 1.78	0.353
12 months	3.60 ± 1.50	4.26 ± 1.29	5.06 ± 1.87	**0.03**
Reduction in PD (mm)	4.13 ± 1.66	3.47 ± 1.73	2.21 ± 2.27	**< 0.001**
*p*-values for intergroup difference(reduction in PD)	**0.001 vs. control** 0.223 vs. delayed GTR	**0.005 vs. control** 0.223 vs. immediate GTR	**0.005 vs. delayed GTR** **0.001 vs. immediate GTR**	

Bold signifies statistical significance. n = sites; PD: pocket depth

### Regression analysis

Table 4 presents the results of regression models to aid in understanding the impact of various variables on IBD reduction at the 1-year postoperative visit. Univariate analyses revealed that infrabony defects with two osseous walls (− 2.89; 95% confidence interval [CI] [− 3.83, − 1.95]; *p* < 0.001) and a combination of infrabony defects (− 1.63; 95% CI [− 2.54, − 0.71]; *p* = 0.001) were significantly associated with less IBD reduction than infrabony defects with three osseous walls. A higher initial IBD (0.73; 95% CI [0.58, 0.88]; *p* < 0.001) and mandible d-M2 infrabony defects (compared to maxilla ones) (1.54; 95% CI [0.62, 2.46]; *p* = 0.002) were significant predictors of increased IBD reduction. However, the timing of GTR surgery (*p* = 0.476) and patient age (*p* = 0.734) did not predict IBD reduction.

**Table 4:** Results of the regression models evaluating the effect of different variables on the changes in the infrabony defect depths (IBDs) of the treated defects at the 1-year recall

Variables	b	S.E	t	β (95% CI)	*p*	m_b	m_S.E	m_t	aβ (95%CI)	ap
Age (years)	−0.01	0.04	−0.34	−0.01 (− 0.08, 0.06)	0.734					
**Initial infrabony defect depth [initial IBD (mm)]**	**0.73**	**0.08**	**9.58**	**0.73 (0.58**,** 0.88)**	**< 0.001**	**0.57**	**0.08**	**7.14**	**0.57 (0.42**,** 0.73)**	**< 0.001**
Occlusion										
Maxilla				0.00 (Reference)					0.00 (Reference)	
**Mandible**	**1.54**	**0.47**	**3.27**	**1.54 (0.62**,** 2.46)**	**0.002**	**0.70**	**0.31**	**2.31**	**0.70 (0.11**,** 1.30)**	**0.025**
Defect morphology										
Three osseous walls				0.00 (Reference)					0.00 (Reference)	
**Combination**	**−1.63**	**0.47**	**−3.47**	**−1.63 (− 2.54**,** − 0.71)**	**0.001**	−0.61	0.36	−1.67	−0.61 (− 1.32, 0.10)	0.100
**Two osseous walls**	**−2.89**	**0.48**	**−6.01**	**−2.89 (− 3.83**,** − 1.95)**	**< 0.001**	**−1.09**	**0.41**	**−2.63**	**−1.09 (− 1.90**,** − 0.28)**	**0.011**
Timing										
Delayed				0.00 (Reference)						
Immediate	−0.36	0.50	−0.72	−0.36 (− 1.35, 0.63)	0.476					

When the significant factors from the univariate models were evaluated in a multivariate model, it was shown that infrabony defects with two osseous walls (− 1.09; 95% CI [− 1.90, − 0.28]; *p* = 0.011) were linked to less IBD reduction. Meanwhile, a greater initial IBD (0.57; 95% CI [0.42, 0.73]; *p* < 0.001) and mandible M2 (0.70; 95% CI [0.11, 1.30]; *p* = 0.025) showed positive correlations with greater IBD reductions.

Table 5 shows the relationship between M2 location and the classification of infrabony defects. The chi-square test showed that maxillary M2s were significantly associated with a smaller residual infrabony defect wall (*p* < 0.001).

**Table 5:** Results of the Chi-square test evaluating the correlation between defect morphology and occlusion

Variable	Total (*n* = 109)	Three osseous walls (*n* = 36)	Combination (*n* = 33)	Two osseous walls (*n* = 40)	Statistic	*p*
Occlusion, n (%)					Χ² = 14.00	**< 0.001**
Maxilla	35 (32.11)	3 (8.33)	14 (42.42)	18 (45.00.00)		
Mandible	74 (67.89)	33 (91.67)	19 (57.58)	33(91.67)		

n = sites

## Discussion

### Clinical and radiographic outcomes of delayed GTR

To evaluate the impact of delayed surgery, this study assessed IBD and PD reductions in d-M2 infrabony defects. Our findings reveal that delayed GTR treatment led to significant reductions in both IBD (2.98 ± 1.98 mm) and PD (3.47 ± 1.73 mm). These findings align with a previous randomized controlled study that reported an average IBD reduction of 3.4 ± 1.2 mm in d-M2 infrabony defects 1 year after delayed GTR treatment using a resorbable polylactic acid barrier, and a reduction of 2.0 ± 1.6 mm 1 year after delayed GTR treatment using a non-resorbable polytetrafluoroethylene barrier [35]. Kim et al. reported a higher IBD reduction in delayed GTR treatment in d-M2. After 4 months, they achieved a 7.57 ± 2.14 mm bone gain. However, their study included patients with a higher initial IBD compared to the present study (10.26 ± 2.59 mm vs. 7.22 ± 2.14 mm). Also, the study period was only 4 months, which was shorter than the duration of our study. It remains unclear whether the radiographs showed new bone formation or only the filling of biomaterial.

This study sought to compare the effect of delayed and immediate GTR treatment for d-M2 infrabony defects. To the best of our knowledge, this is the first investigation to uncover the effects of different d-M2 GTR treatment timeframes, making direct comparisons with previous research challenging. Both test groups exhibited significant IBD reductions (Immediate GTR group, 2.77 ± 1.97 mm; Delayed GTR group, 2.98 ± 1.98 mm; Control group, 0.68 ± 1.03 mm; *p* < 0.001) and PD reductions (Immediate GTR group, 4.13 ± 1.66 mm; Delayed GTR group, 3.47 ± 1.73 mm; Control group, 2.21 ± 2.27 mm; *p* < 0.001) compared to the control group. These findings are consistent with those of previous research reporting a mean PD reduction of 3.32 ± 0.62 mm 1 year after immediate GTR treatment [36]. However, the current study did not reveal significant differences in bone regeneration between the groups receiving delayed and immediate GTR treatments (*p* = 0.84). Moreover, regression analysis indicated that the timing of GTR treatment was not a prognostic factor for d-M2 GTR outcomes (*p* = 0.476). Since there were no differences in the effects of delayed and immediate GTR treatment in d-M2 infrabony defects, for patients requiring GTR treatment and M3 extraction, immediate GTR may be more beneficial considering the single required surgical procedure compared to multiple procedures performed in patients receiving delayed GTR treatment.

However, when GTR treatment was used for other sites, some studies reported higher reductions in PD and IBD. Cotellini et al. found that using an e-PTFE membrane achieved a 5.9 ± 2.5 mm PD reduction one year after treatment [37]. Gorski found that using a collagen membrane plus a xenogenic graft resulted in a 4.4 ± 1.8 mm IBD reduction one year after treatment [38]. The site difference may contribute to this variation. Mikami et al. found that molars were significantly associated with less PD reduction compared to incisors [39]. Therefore, a deeper investigation into the prognostic factors affecting GTR treatment in d-M2 infrabony defects is needed.

### Prognosis factor for regenerative outcome in d-M2 infrabony defects

The positioning of M2 at the posterior part of the oral cavity presents unique challenges for GTR treatment compared to treatment at other oral sites. Identifying patient- and defect-related factors that influence treatment outcomes becomes crucial for improving the predictability and effectiveness of GTR-treated d-M2 infrabony defects. To the best of our knowledge, this study is the first to investigate the prognostic factors affecting GTR treatment in d-M2. In this study, the initial IBD was found to have a positive effect on the bone fill achieved through GTR treatment of d-M2 infrabony defects. This observation aligns with details of previous reports [40–42]. Mikami et al. reported that the IBD at baseline affected clinical outcomes in the treatment of infrabony defects in a three-year retrospective study, indicating that a deeper initial IBD leads to more favorable results in terms of infrabony defect reduction [40].

Another factor impacting the 1-year postsurgical outcomes was the morphology of the defect. Defect morphology has been shown to influence the clinical outcome of periodontal regenerative therapy. Aoki et al. demonstrated that defect morphology (3-wall or 1-2-wall defect) significantly affected the amount of radiographic bone fill two years after periodontal regeneration therapy [43]. However, previous studies have not reported on the prognostic factors that might influence bone regeneration after periodontal regenerative therapy in d-M2 infrabony defects. The present study showed that infrabony defects with two osseous walls were a significant prognostic factor for IBD reduction at the one-year examination, which is consistent with earlier findings. A previous meta-analysis reported that fewer remaining walls in these defects correlate with reduced bone regeneration and clinical attachment level, supporting our results [42]. However, the present study has a limitation: the classification may not accurately portray defect morphology in the immediate GTR group. Wider infrabony defects were observed in this subgroup due to an extracted M3 socket, potentially influencing the effectiveness of GTR treatment [44, 45]. Future studies should aim to achieve a more precise classification or to record additional parameters to develop a comprehensive depiction of defect morphology in immediate GTR-treated d-M2 infrabony defects.

Recent studies indicate that maxillary M3s increase the risk of d-M2 infrabony defects, and these defects are more severe in the maxilla than in the mandible [23]. Despite this, investigations of GTR treatment for maxillary d-M2 infrabony defects are lacking. Hence, in this study, the second molar (M2) location was included and analyzed. Table 4 shows that the mandible exhibits more favorable bone fill compared to the maxilla, possibly due to the tendency of the maxillary M2 to form two wall infrabony defects. Table 5 further supports this result by demonstrating a significant association between maxillary M2 and the reduction in residual infrabony defect walls. Previous research has also reported lower bone density in the maxillary molar area compared to the mandibular area [46]. After tooth extraction, contour loss occurs within the first 6 months, particularly in the maxillary bone, which may explain these observations [24]. However, due to the retrospective nature of this study, our findings represent only an initial exploration of the impact of M2 location on d-M2 GTR outcomes. Future research using a randomized controlled design will be essential to provide clearer insights into this topic.

Finally, despite the variations recorded in previous studies [47], our study did not observe a significant correlation between patients’ age and IBD reduction 1 year after GTR treatment, which aligns with the findings of other studies [48]. In our study, the mean age was 40.00 ± 8.26 (range, 27–67) years, and all patients were required to be > 18 years of age for enrollment. Earlier research has indicated age to be a factor influencing the healing of d-M2 infrabony defects after M3 extraction [13], but our study noted a considerable increase in bone fill in both GTR-treated groups. This observation is consistent with a previous study that found aging was not associated with IBD reduction after 1 year in GTR-treated teeth [39].

Although significant clinical and radiographic improvements were observed in both GTR groups, the results of this study should be interpreted with caution. First, despite efforts to standardize treatment approaches, the study has the inherent limitation of a retrospective observational design. Second, due to limited information, soft tissue data, third molar impaction data, and patient-reported outcomes were not included. Additionally, no suitable classification criteria were available to assess the impact of socket morphology on infrabony defects after third molar extraction, which affects the reproducibility of these measurements. Furthermore, the suture materials varied among patients. Therefore, well-designed, bias-controlled clinical trials are needed to apply our findings in clinical practice and draw reliable conclusions.

## Conclusion

We examined whether delayed or immediate GTR treatments statistically improve the reduction in IBD in d-M2 infrabony defects. Ultimately, there was no statistical difference in results between delayed and immediate GTR treatments. Hence, immediate GTR is likely more beneficial to patients due to requiring fewer surgical procedures. The morphology of infrabony defects, the M2 location, and the initial depth of infrabony defects all appear to affect the outcomes of GTR in d-M2 infrabony defects.

## Data Availability

Data availability-The data supporting this study’s findings are available from the corresponding author upon reasonable request.

## Abbreviations

D-M2: Distal aspect of the second molar
GTR: Guided tissue regeneration
M3: Third molar
IBD: Infrabony defect depth
SRP: Scaling and root planing
PD: Pocket depth
M2: Second molar
BOP: Bleeding on probing
CBCT: Cone beam computed tomography
DBBM: Deproteinized bovine bone material
CM: Collagen membrane
CEJ: Cemento-enamel junction
BD: The base of the defect
MPR: Multi-Planar Reconstruction
A-M3: Adjacent third molar
CI: Confidence interval
